# Electrocatalytic CO_2_ Reduction Coupled to Formate Fermentation: An Electro‐Bio Cascade Approach in Biocompatible Electrolytes

**DOI:** 10.1002/chem.202502658

**Published:** 2025-12-24

**Authors:** Luciana Vieira, Jonathan Thomas Fabarius, Gabriela Rizzo Piton, Barbara Bohlen, Dhananjai Pangotra, Melanie Speck, Carina Sagstetter, Volker Sieber, Arne Roth

**Affiliations:** ^1^ BioCat Branch for Bio, Electro and Chemocatalysis Fraunhofer Institute for Interfacial Engineering and Biotechnology (IGB) Straubing Germany; ^2^ TUM Campus Straubing For Biotechnology and Sustainability Technical University of Munich Straubing Germany

**Keywords:** biomass growth, carbon dioxide, electrochemical CO_2_ reduction, formic acid, microbial C1 fermentation

## Abstract

Integration of electrochemical CO_2_ reduction with microbial fermentation enables conversion of CO_2_ into valuable chemicals but poses challenges at the electrolysis‐fermentation interface. The electrolyte must ensure efficient CO_2_ reduction while remaining compatible with microbial growth. We investigated various electrolytes for coupling CO_2_ electroreduction to formate with formate fermentation by *Methylorubrum extorquens* TK 0001. Electrolyte performance was evaluated by formate production and microbial growth. A phosphate‐based buffer demonstrated the best overall compatibility. Optimal microbial growth occurred at 0.1 mol L^−1^ KPi, with tolerance of up to 111 mmol L^−1^ formate. Continuous CO_2_ electrolysis in 1.0 mol L^−1^ KPi produced 2.0 mol L^−1^ formate in 48 h. Formate fermentation with *M. extorquens* showed biomass yield of 107 mg CDW g_formate_^−1^ and a growth rate of 0.10 h^−1^. These results highlight the crucial role of buffer composition and concentration in balancing efficient CO_2_ electroreduction with stable fermentation. Optimizing this electrochemical–biological interface enables direct utilization of CO_2_‐derived formate as a substrate for sustainable microbial production, offering a promising scalable route for industrial biotechnology.

## Graphical abstract

CO_2_ utilization in industrial biotechnology: Rational design of a process cascade for electrochemical CO_2_ reduction linked with formate fermentation, supporting robust cell growth and high biomass yields.

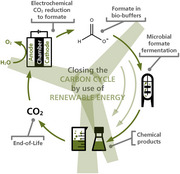

## 1 Introduction

The rising levels of carbon dioxide (CO_2_) in the atmosphere are the primary contributor to global warming and among the most pressing modern challenges [1, 2]. In reply to this challenge, there is an urgent need to transition away from our conventional linear economy model. This current model relies on extracting fossil carbon feedstock from the ground, which is emitted as CO_2_ into the atmosphere. Instead, a circular and sustainable carbon economy based on renewable resources has to be implemented to achieve a closed carbon cycle and to stop adding fossil carbon into the environment. The urgency for this transition has driven the development of various sustainable processes targeting net‐zero carbon emissions [3, 4]. In this context, CO_2_ capture and utilization (CCU) approaches emerge as an essential technology pillar, enabling a circular carbon economy by transforming CO_2_ from a climate‐damaging waste stream into a valuable raw material for the sustainable production of chemicals and fuels [2, 5].

Among several catalytic methods for CO_2_ utilization, electrocatalytic CO_2_ reduction (eCO_2_R) is particularly promising [2, 6]. Powered by renewable electricity, this process enables CO_2_ activation under mild conditions and allows direct hydrogenation of CO_2_ on electrode surfaces without requiring the application of pressurized hydrogen gas [7, 8]. By adjusting the reaction parameters, such as electrode material, electrolyte composition, and pH, eCO_2_R can selectively produce industrially relevant C_1_ and C_2_ compounds, including carbon monoxide (CO), formic acid (HCOOH), and ethylene (C_2_H_4_), with high production rates [9, 10]. While the sole application of eCO_2_R at high conversion rates remains challenging for synthesizing complex chemical compounds (i.e., C_3+_), it is possible to explore whole‐cell biocatalysis and their broad product spectrum to upgrade eCO_2_R products to high value‐added compounds [11, 12, 13].

In this context, particularly the microbial utilization of C_1_ compounds like methanol and formic acid/formate received attention as appealing option to utilize CO_2_‐derived substrates in biotechnology [11, 14]. Microorganisms capable of valorizing such substrates are classified as methylotrophs and formatotrophs, respectively [15]. Compared to conventional host microbes in industrial biotechnology, consuming glucose, methylo‐/formatotrophic microorganisms provide a highly specialized metabolism to utilize and deal with harsh substrates like formate/formic acid and their corresponding toxic metabolites like formaldehyde [16]. Thus, integrating eCO_2_R with subsequent biochemical or chemical steps in cascade processes opens pathways to more complex and valuable chemicals.

Among the various products of CO_2_ electroreduction, formic acid emerges as a promising liquid intermediate for integration with biochemical processes. Compared to gases, such as CO_2_ and CO, its easy handling and high solubility in aqueous media provide a significant advantage when used as substrate in fermentation [17]. With formic acid as CO_2_‐derived liquid and water‐soluble substrate, several challenges associated with traditional gas fermentation, such as low gas‐to‐liquid mass transport rates and highly specialized equipment required for handling gaseous (and, in case of hydrogen present, explosive) feed streams, are solved [12, 13]. Moreover, the chemical stability of formate makes it an ideal platform chemical for biological transformations.

Formate is metabolized by native formate‐utilizing microorganisms and metabolically engineered synthetic formatotrophs [11, 18]. Synthetic biology and metabolic engineering significantly enabled the application of synthetic formatotrophs for selective formate conversion into biomass with respectable yields of up to 100 mg_cell dry weight_ g_formate_^−1^ and also into high‐value chemicals [11, 18, 19, 20, 21, 22].

Various microbial metabolic pathways are connected to C1 assimilation. The C1 pathway architecture generally depends on the host but is limited to six main pathways. In the case of formic acid, direct utilization is possible by the Wood–Ljungdahl pathway, the reductive glycine pathway, or the Serine cycle. We selected a major Serine‐cycle model methylotroph, that is, *Methylorubrum extorquens *TK 0001, due to its well‐understood metabolism, established cultivation techniques, known fermentation strategies, and broad availability of genetic engineering tools for this strain [23, 24].

Several approaches have been suggested for integrating eCO_2_R with biological transformations. Described are conventional approaches integrating bio‐electrochemical reactors, that is, the electrochemical and the biochemical reactions taking place in a single reactor, and the spatial decoupled approach with two independent reactors [25, 26, 27, 28]. Conventional approaches, often referred to as microbial electrosynthesis (MES) or electro‐fermentation, rely primarily on anaerobic microbes directly attached to the cathode or anode surface or submerged cells to harvest or discharge electrons, respectively [25, 29]. Significant challenges arise, such as low current densities, low media concentrations, and low conductivity. Furthermore, only a limited number of electro‐active microbial strains are available. Therefore, MES studies are often restricted to acetate production as electro‐active microbes have a limited product spectrum [30].

On the other hand, spatial decoupling of eCO_2_R and biological transformation has emerged as a more efficient alternative to tackle the previously described challenges. It can achieve higher conversion rates and take advantage of high substrate concentration in electrolytes [31]. Furthermore, it opens new opportunities for aerobic fermentation and the broad product portfolio usually associated with O_2_‐dependent microorganisms [32].

Following this direction, Stöckl et al. proposed a decoupled semi‐automated fed‐batch reactor, reaching higher current densities (150 mA cm^−2^) and employing high‐concentration electrolytes (0.2 mol L^−1^). In their work, formate electrosynthesis was evaluated at different phosphate‐buffer systems containing only Na^+^ or K^+^ ions or a mixture of them. The phosphate buffer system containing only K^+^ ions yielded higher formate concentrations and stable faradaic efficiency (FE) over 22 h of electrolysis. The authors attributed the improved performance to the higher conductivity of the potassium‐based phosphate buffer solution. However, when the buffer systems were inoculated with *Cupriavidus necator* (wildtype, resting cells) targeting polyhydroxybutyrate (PHB) production, the higher PHB concentration was achieved with Na^+^/K^+^ mixture phosphate‐buffer, reaching up to 43.8 ± 3.0 mg L^−1^ OD^−1^[33].

In another work, Lim et al. [26]. attempted to integrate eCO_2_R to formate with microbial fermentation of *C. necator* to PHB in a so‐called biohybrid reactor. Initially, a flow cell reactor for converting CO_2_ to formate was inoculated with *C. necator* and an MR medium as the electrolyte solution. However, cells did not survive due to interaction with the cathode and possible reactive species generated at the anode. Therefore, a two‐reactor system was proposed. In the new design, electrolyte solution and fermentation broth were circulated between the electrolytic and the fermentation reactors with an intermediate filtration step to retain the cells in the fermentation tank. Further challenges were the low electric conductivity of the medium and electrode poisoning by the presence of trace metals in the buffer, as, at negative potentials, metal deposition at the cathode occurred. Such limitations were addressed by electrolyte optimization. First, electrolyte conductivity was addressed by increasing phosphate salt concentration (30 g L^−1^ of KH_2_PO_4_ was added); second, since metal ions from the trace metal solution (TMS) were being deposited at the cathode, the TMS was removed from the medium. Precipitates at the cathode surface were identified by XRD analysis as struvite (MgNH_4_PO_4_.6H_2_O) and KMgPO_4_·6H_2_O. Therefore, nitrogen salt was removed from the medium, and Mg^2+^ concentration was limited to 0.1 g L^−1^. Additionally, PHB accumulation increases under nitrogen‐limited conditions [34].

A recent study by Zhang and coworkers [35] demonstrated an integrated and continuous electro‐microbial conversion with C_2_ intermediates for the biosynthesis of PHA (polyhydroxyalkanoates) using *Pseudomonas putida*. Electrolyte compatibility was investigated with carbonate buffer, widely used in electrosynthesis, and a “basal” solution containing mainly phosphate buffer. Even though pH was controlled and maintained at 7.2, *P. putida* showed only minimal growth in carbonate buffer, demonstrating the preference of the strain for a phosphate‐containing medium. Although copper catalysts are broadly investigated for CO_2_ conversion to C_2_ products and these are preferred microbial substrates [36], they still lack selectivity toward a single product. In the work by Zhang et al., ethanol and total soluble C_2+_ FE did not exceed 25 %. The lower efficiency of eCO_2_R to C_2+_ products inspires synthetic biologists to improve microbial C_1_ uptake, as these feedstocks are produced with higher efficiency from eCO_2_R.

Despite the advances achieved in the reported studies, a systematic understanding of the relationship between buffer composition, electrochemical performance, and robustness of microbial fermentation remains unexplored. In this study, we address this gap by examining the electrochemical performance of eCO_2_R to formate in various biological buffers and their biocompatibility with *M. extorquens*. We explored parameters such as pH stability, current density, electrode stability, and formate productivity for five buffer compositions, with the target to optimize conditions for integration with biocatalytic processes.

Furthermore, we demonstrated the viability of using electrochemically produced formate to enable *M. extorquens* growth in a phosphate buffer system without any intermediate purification steps. This study highlights the potential of using CO_2_‐derived formate as a feedstock for biotechnological applications and thus contributes significantly to the development of process cascades combining CO_2_ utilization and biotechnology.

## 2 Results and Discussion

The successful integration of electrochemical CO_2_ reduction with microbial fermentation requires careful consideration of medium conditions that can support both processes. We evaluated five biological buffers as potential electrolytes for eCO_2_R: potassium phosphate (KPi), sodium carbonate/bicarbonate (NaCBi), tris(hydroxymethyl)aminomethane (TRIS), citrate (CPi), and potassium bicarbonate (KHCO_3_). Each buffer was prepared at a concentration of 1 mol L^−1^ to ensure sufficient conductivity, minimize cell resistance, and overall cell voltage. To reduce other effects of present compounds that may interact with the growth of *M. extorquens* to a minimum, a mineral salts medium without any supplied vitamins or complex components was used as the basic growth medium. CO_2_ electrolysis in different buffers was conducted under galvanostatic conditions at −100 mA cm^−2^ using a flow cell setup.

### 2.1 Biologic Buffers Enable eCO_2_R Integration With Microbial Fermentation

**FIGURE 1:**
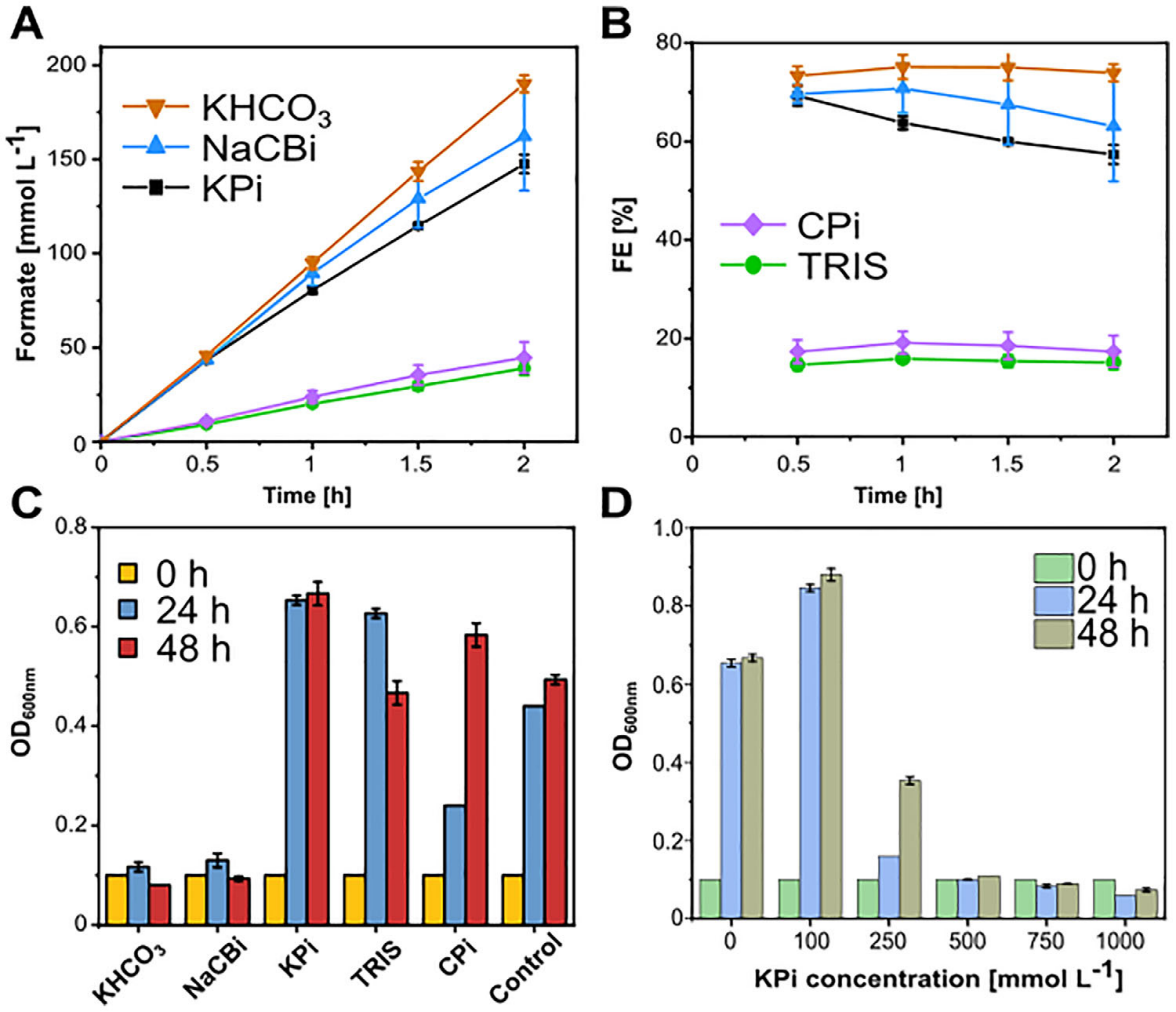
Buffer electrolyte screening for electrochemical and biological applications. (A) Formate concentration, (B) Faradaic efficiency, (C) Optical density (OD_600_) measured at 600 nm using samples of grown cells in MO media supplemented with 0.1 mol L^−1^ of different buffer solutions at 0 h, 24 h, and 48 h, (D) Optical density (OD_600_) measured at 600 nm using samples of grown cells in MO medium supplemented with varying KPi buffer concentrations. Electrolysis experiments were conducted in two independent replicates, biological experiments in triplicates; error bars reflect standard deviations.

The electrochemical performance revealed a clear distinction between inorganic and organic buffers (Figure 1 A,B), which led to different formate concentrations, FEs, and conductivities. The inorganic electrolytes (KHCO_3_, NaCBi, and KPi) demonstrated superior performance, with KHCO_3_ achieving the highest formate concentrations of 190 mmol L^−1^ and FE of nearly 80 % over a 2‐hour electrolysis period. In contrast, the organic buffers TRIS and CPi showed limited productivity, with formate concentrations remaining below 50 mmol L^−1^ and FE of 17 % for both electrolytes. These results align with the reported suitability of KHCO_3_ as an effective electrolyte in eCO_2_R [6, 37, 38, 39], while NaCBi also shows promising performance.

The superior performance of inorganic buffers correlates strongly with the electrical conductivity of their respective solutions. At concentrations of 1.0 mol L^−1^, KHCO_3_, NaCBi, and KPi solutions exhibited electric conductivities above 50 mS cm^−1^, with KPi reaching the highest value of 100 mS cm^−1^. In contrast, at the same concentration, TRIS and CPi solutions showed significantly reduced conductivities below 40 mS cm^−1^. This difference in conductivity directly impacts the system's ability to efficiently carry current during electrolysis, affecting both the overall cell voltage and energy efficiency (see Supplementary Information Figure S1) of the process [40]. In addition, the advantageous effect of cations such as Na^+^ and K^+^ on eCO_2_R is an aspect missing in organic buffers. In particular, K^+^ cations have been reported to play a role in stabilizing a key intermediate species (*COO^−^) during CO_2_ conversion to formate [41, 42].

The biological compatibility of these electrolytes was assessed by evaluating *M. extorquens* cell growth in minimal media supplemented with each buffer at 0.1 mol L^−1^. Our results revealed an interesting trade‐off between electrochemical and biological performance (Figure 1C). Despite their superior electrochemical performance with FE of 80 % and 65 %, respectively, KHCO_3_ and NaCBi significantly inhibited cell growth, as evidenced by the low optical density measurements at 600 nm, maintaining constant over 48 h. Conversely, TRIS and CPi, which showed modest electrochemical performance (FE of 17 % and 15 %), supported cell growth comparable to or exceeding the control medium without buffer addition. Notably, TRIS demonstrated exceptionally high cell growth after 24 h, but cell density decreased after 48 h. On the other hand, on KPi solution, final optical density of 0.67 was achieved. Hence, KPi emerged as the optimal compromise, maintaining reasonable electrochemical performance (60 % FE at 100 mA cm^−2^ for 2 h) while supporting the highest cell growth among all evaluated conditions.

Given KPi's outperformance, we conducted a detailed investigation of *M. extorquens* growth response to varying the buffer concentrations, ranging from 0 (minimal medium only) to 1.0 mol L^−1^, with growth monitored at 0, 24, and 48 hours (Figure 1D). The results revealed optimal cell growth at 0.1 mol L^−1^ KPi, with higher concentrations showing progressive growth inhibition, with complete growth suppression observed at concentrations above 0.5 mol L^−1^. These findings establish clear operational boundaries for our integrated system: the electrochemical phase requires 1.0 mol L^−1^ KPi for optimal performance, necessitating a subsequent 10‐fold dilution for the biological optimum operation at 0.1 mol L^−1^ KPi. This dilution requirement prompted the investigation of two critical aspects: the maximum achievable formate concentration in long‐term electrolysis using 1.0 mol L^−1^ KPi and the upper limits of microbial formate tolerance.

### 2.2 Enhanced Formate Concentration via eCO_2_R in Potassium Phosphate Buffer

**FIGURE 2:**
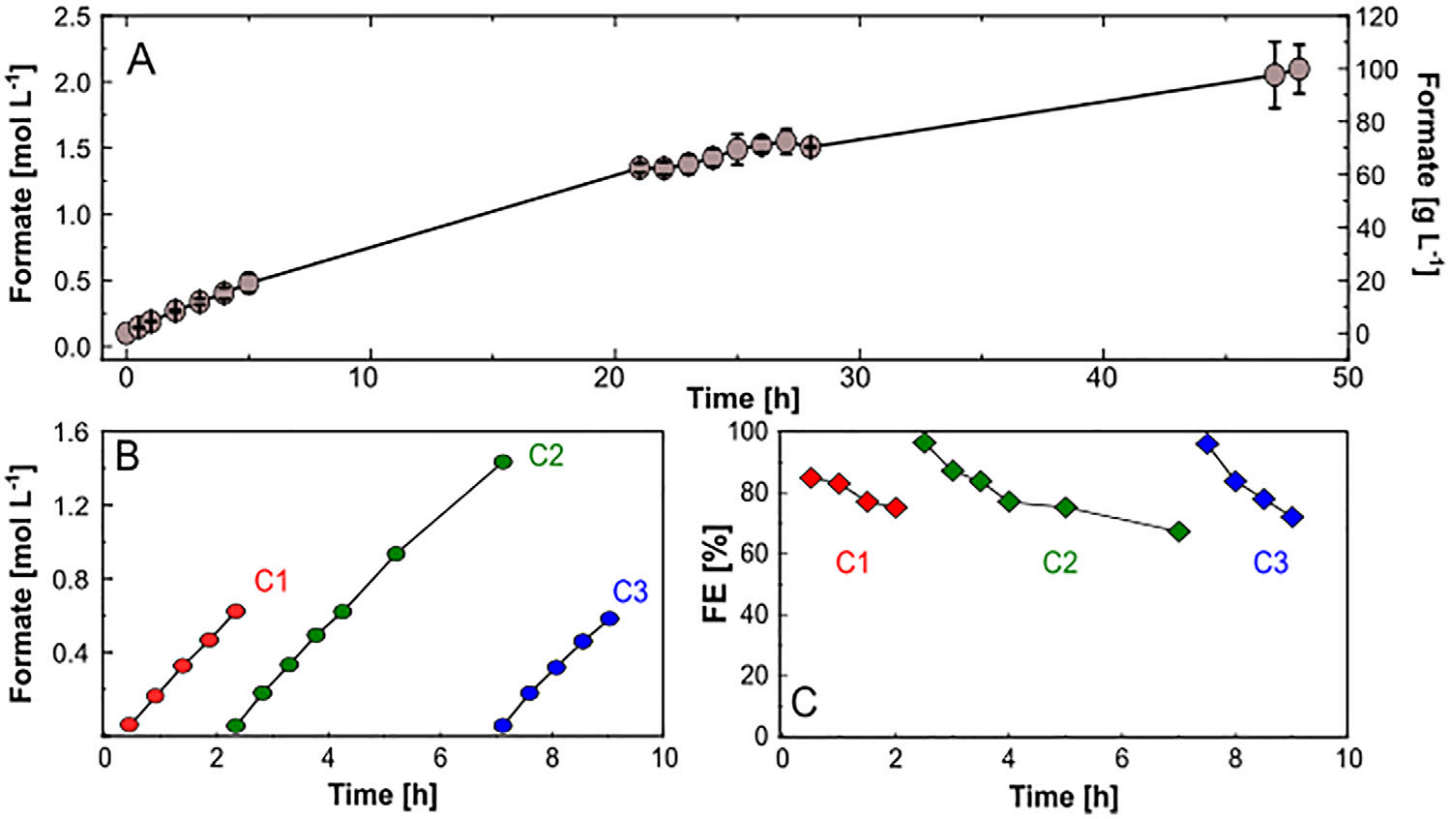
Long‐term CO_2_ electrolysis. (A) Continuous eCO_2_R in 1 mol L^−1^ KPi buffer at 100 mA cm^−2^ for 48 h (*n* = 4). (B) Cyclic operation involving sequential cycles of 2 and 4 h in 1 mol L^−1^ KPi buffer at 100 mA cm^−2^. The experiments were performed in a flow cell with recirculation mode with an electrolyte flow rate of 100 mL min^−1^. CO_2_ was supplied to the gas diffusion Sn‐coated cathode with a flow rate of 100 mL min^−1^. Data were acquired in quadruplicates (error bars reflect standard deviation) (A) and individual experiments (B and C).

Having identified KPi as the optimal buffer, we investigated the long‐term performance for formate production. Therefore, extended electrolysis experiments were conducted in quadruplicates at an optimized current density of 100 mA cm^−2^ (refer to Figure S2) in 1.0 mol L^−1^ KPi to evaluate maximum achievable formate concentrations (Figure 2A). The system demonstrated remarkable productivity and high reproducibility, surpassing formate concentrations of 2.0 mol L^−1^ (100 g L^−1^) over 48 h of continuous operation under recirculation mode. This represents the highest formate concentration reported using biological buffers for CO_2_ electroreduction (see Table S3 in the Supplementary Information). Despite this impressive productivity, we observed a gradual decline in system performance, with FE decreasing from initial values of 80 % in the first 10 h to approximately 30 % by the end of the experiment (Figure S3). Similarly, the formate production rate decreased from an initial 1.8 mmol h^−1^ cm^−2^ to below 0.8 mmol h^−1^ cm^−2^ after 48 h.

**FIGURE 3:**
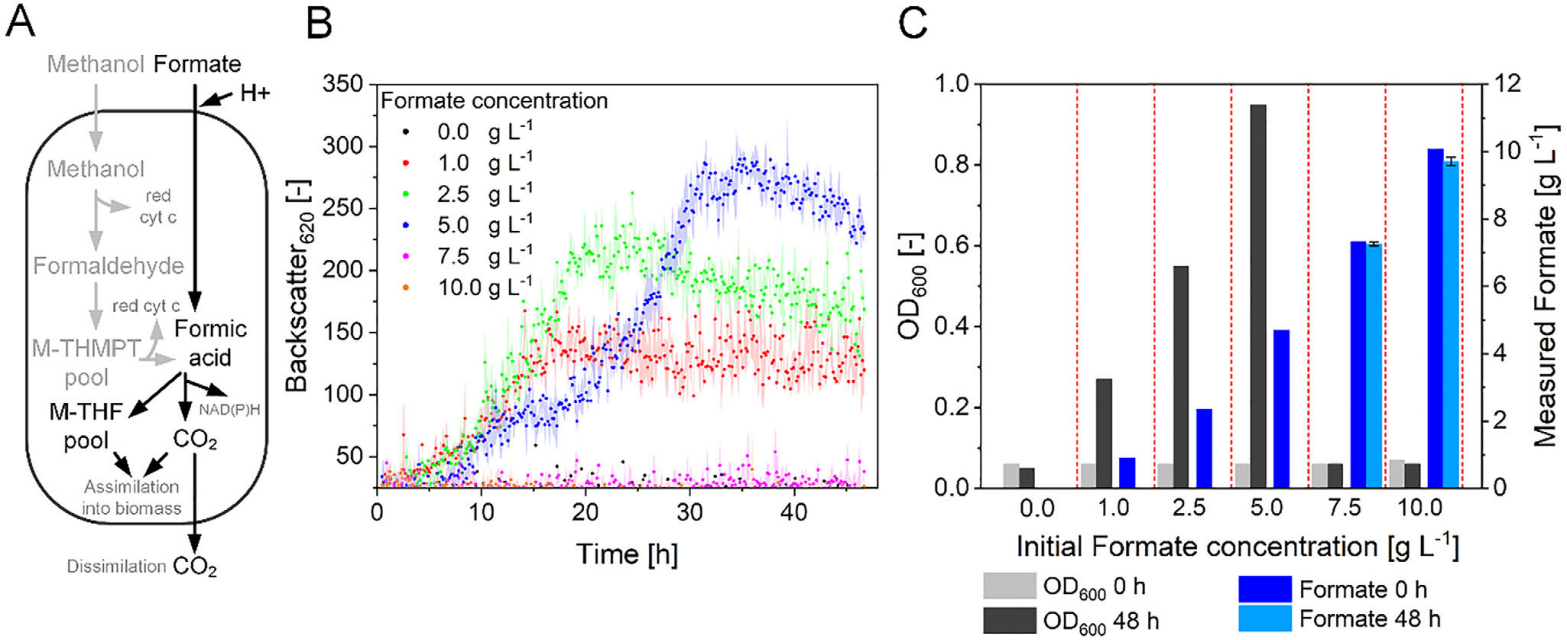
Formate tolerance of *M. extorquens* TK 0001 in microbioreactor cultivations. (A) Overview of the metabolic C_1_ assimilation routes in *M. extorquens*. Formate/methanol is oxidized to generate redox power (e.g., red cyt c and NAD(P)H by dissimilation via formate dehydrogenase into CO_2_). It is assimilated into biomass from the M‐THF pool. Abbreviations: red cyt c, reduced cytochrome‐c; nicotinamide adenine dinucleotide (phosphate)‐reduced, NAD(P)H; M‐THF, methylenetetrahydrofolate. (B) Growth profiles of *M. extorquens* biomass measured at 620 nm backscatter in the pH‐stat BioLector experiments applying formate concentrations from 0 to 10 g L^−1^ and pH titration using 2 mol L^−1^ H_2_SO_4_ and 3 mol L^−1^ NaOH. (C) Correlation between obtained OD_600_ after 48 h of cultivation with supplied (0 h) and residual (48 h) formate concentration. The cultivations were carried out in biological triplicates; error bars reflect standard deviations.

Considering the integration with the biosynthesis process, we also monitored the catholyte's and anolyte's pH during the 48 h electrolysis (Figure S4). Overall, the catholyte pH increased to almost 9.0 over the first 4 h, but with the accumulation of formate in the solution, the pH steadily decreased to approximately 6.5 after 48 h. The solution pH plays a crucial role in biosynthesis, and specifically for *M. extorquens*, growth is inhibited at pH values higher than 8.0 [20]. Considering that the pH of the media also increased with formate consumption due to uptake of the protonated form (i.e., formic acid) by *M. extorquens* (Figure 3A), external pH control during the fermentation is necessary to maintain optimal conditions.

The decrease in process performance, reflected in the loss of FE, is often associated with the failure of the GDE [43]. Although the GDE structure contains a hydrophobic component, over time, water tends to infiltrate through the GDE structure, limiting its efficiency due to flooding of the gas compartment. Another common factor is the increased local pH at the electrode/electrolyte interface during the eCO_2_R reaction, favoring carbonate species formation. Finally, the carbonate concentration will exceed its maximum solubility in water, and carbonate salts will deposit at the electrode surface, completely blocking active catalytic sites and leading to electrode inactivation. Reasons leading to GDE failure due to carbonate deposits were investigated by scanning electron microscopy (SEM) coupled with energy‐dispersed X‐ray spectroscopy (EDX) of the working electrode before and after electrolysis. Before electrolysis, SEM images exhibited a homogeneous coverage of the catalyst layer on the GDE surface (Figure S5A). However, after 24 h electrolysis, the electrode surface was observed to be covered by crystals (Figure S5B), explaining the FE loss observed after prolonged electrolysis. Further EDX analysis (Figure S6 A,B) indicates the composition of the salt deposits as potassium and phosphorus‐based, in agreement with the composition of the electrolyte solution. The partial recovery of FE in the CO_2_ electrolyzer has been reported by flushing the cathode compartment with water to resolubilize the carbonate deposited at the electrode surface [44, 45]. To overcome the limitations of GDE flooding and local pH increase, we developed a cyclic operation strategy (Figure 2B–C) involving sequential electrolysis cycles. The cathode compartment was circulated with pure water between cycles, and a fresh electrolyte solution was used at each cycle. This approach maintained the FE above 70 %, with each 2‐hour cycle producing approximately 0.650 mol L^−1^ formate and the 4‐hour cycle achieving 1.4 mol L^−1^, a considerable improvement in time compared to the continuous 48‐hour electrolysis shown in Figure 2A.

### 2.3 Evaluating Formate Toxicity on *M. extorquens* TK 0001

The high formate concentrations achieved with the optimized system demonstrate its potential for scalable electrochemical formate production. Yet, successful integration with biological processes, for example, the development of a feeding strategy to avoid substrate inhibition, requires careful consideration of microbial substrate tolerance and rate compatibility of the two processes.

Therefore, we examined the formate toxicity in the BioLector using pH‐stat conditions and neutralized potassium formate solutions. In particular, cell growth response to varying initial formate concentrations in batch fermentations ranging from 0 to 10 g L^−1^ was examined (Figure 3B–C).

As expected, no growth was observed when no carbon source (0 g L^−1^ formate) was added to the growth media (Figure 3B–C). The growth kinetic was similar when supplying 1 or 2.5 g L^−1^ of formate and showed only differing final biomass concentrations (Figure 3B). After 48 h, a final OD_600_ value of 0.27 and 0.55 matched the roughly doubled carbon available when 2.5 g L^−1^ formate was supplied (Figure 3C). The growth plots for both conditions indicate formate depletion after 16 and 20 h (Figure 3B), as was confirmed by HPLC measurements (Figure 3C). However, when supplying 5 g L^−1^ of formate, a lag phase of about 20 h was observed. This lag phase indicates adaptation of the strain toward the increased initial formate concentration as a sign of reaching a critical concentration range, starting to become toxic. Interestingly, the final biomass concentration after 48 h was again almost doubled compared to the 2.5 g L^−1^ formate condition (OD_600_ of 0.95 vs. 0.55), with the supplied formate completely consumed after 48 h in both cases (Figure 3C). These data suggest a specific biomass yield of about 55 mg_CDW_ per gram of supplied formate, using the OD_600_‐Cell Dry Weight (CDW) correlation used in our laboratory [19].

Finally, no cell growth was observed above 5 g L^−1^ of supplied formate, and offline measurements showed no change in the inoculation OD_600_ and the supplied formate concentration (Figure 3C). This finding clearly indicates a growth‐inhibiting effect of formate on *M. extorquens *TK0001 at concentrations exceeding 5 g L^−1^ under pH‐stat conditions in a microbioreactor.

The toxicity of formate on microbial growth has been reported before [11]. Previously, we have shown that pH titration significantly improves formate utilization of *M. extorquens* [20]. In summary, the formate tolerance of *M. extorquens* and its native capability to grow on formate render this strain suitable for integration with eCO_2_R. However, the tolerance compared to yeast is limited and only comparable to *E. coli*. To overcome this limitation, the *M. extorquens* formate tolerance can be enhanced by using Adaptive Laboratory Evolution in future studies [46]. The obtained titers of about 100 g L^−1^ achieved with our eCO_2_R system, together with the required buffer dilution, enable direct use of formate from eCO_2_R in *M. extorquens* batch fermentation or usage of the electrolyte as feed solution in fed‐batch processes. This integration in lab‐scale bioreactors was addressed as a next step.

### 2.4 Rational Process Integration of eCO_2_R With Formate Fermentation

**FIGURE 4:**
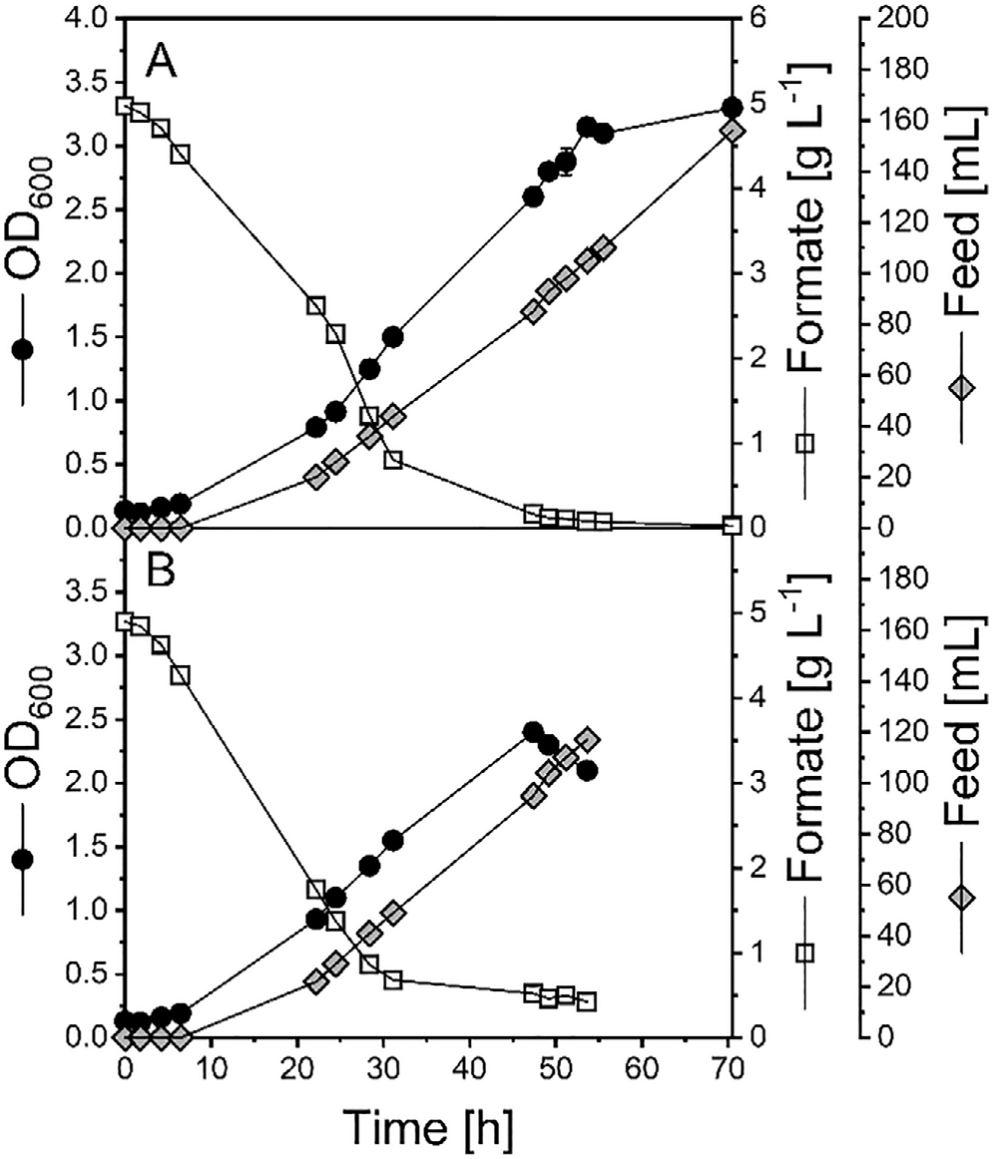
Formate fermentation process development with *M. extorquens* TK 0001. Comparison of 2 L scale fed‐batch fermentations in minimal media with commercial potassium formate (A) and potassium formate generated electrochemically from CO_2_ (B). Data acquired in individual experiments.

With the selected buffer and the knowledge about formate tolerance, we established a bioprocess at 2 L scale using commercial (Figures 4A and S7A) or electrochemically (through eCO_2_R) derived formate in 1.0 mol L^−1^ KPi (Figures 4B and S7B) as the sole carbon sources.

When commercial formate was supplied, the cells grew early in the batch phase and rapidly consumed the substrate. Thereby, a remarkably high substrate uptake rate of 1.55 g_formate_ g_CDW_^−1^ h^−1^ was observed and the feed was already started to avoid carbon depletion. In batch phase, a Y_X/S_ of 54 mg_CDW_ g_formate_^−1^ was calculated, matching perfectly the previously derived Y_X/S_ from the BioLector experiments, which was almost doubled to 101 mg_CDW_ g_formate_^−1^ in the feed phase (Figure S5A). Therefore, the applied limited carbon availability improved clearly the substrate to biomass conversion efficiency.

When eCO_2_R‐formate was used for fermentation, again a rapid consumption of the carbon source in batch phase was observed (Figure 4B). Remarkably, the growth rate was increased by 12 % compared to commercial formate. The Y_X/S,batch_ was with 52 mg_CDW_ g_formate_^−1^ in a similar range compared to the process using commercial formate (Figure S5B). In contrast, the biomass yield in the feed phase was reduced by 22 % when using electrochemically produced formate.

Overall, the performance of the fermentation process utilizing commercial and electrochemically derived formate was found to be quite similar. Only the final maximum achieved biomass concentration was reduced by 27 % when eCO_2_R formate was used (0.73 g_CDW_ L^−1^). This is in line with the likewise reduced biomass yield in the feed phase. Moreover, a drop of the OD was observed in this process (Figure 4B), possibly due to cell lysis or biofilm formation (cells sticking at the reactor wall and thus reducing biomass concentration in solution) as a consequence of high phosphate concentrations introduced with the eCO2R‐formate feed. The effect of phosphate on the fermentation performance is examined further below.

Clearly, the conversion efficiency of the carbon into biomass was increased by applying a feed phase. When both substrates were juxtaposed, it was found that the application of eCO_2_R‐derived formate led to a reduced Y_X/S_ in the feeding phase. The similar Y_X/S_ in both batch phases and the reduced Y_X/S_ in the fed‐batch phase on eCO_2_R formate suggest the presence of a growth inhibiting compounds in the eCO_2_R formate feeding solution. Since both feeding solutions should have similar compositions, the hypothesis was drawn that deviations in the phosphate concentrations or particles from the electrode (e.g., Sn) mainly influence the growth. Such deviations may occur from the conditions in the electrochemical process (e.g. liquid evaporation and electrode leaching). This hypothesis is supported by recent findings, *i.a*., for *M. extorquens* AM1, that increased phosphate concentrations inhibit growth significantly [47] and is in accordance with our observation regarding strong detrimental effect of KPi concentration on the biomass yield (Figure 1D).

### 2.5 Reducing Phosphate Concentration Improves Formate Fermentation

**FIGURE 5:**
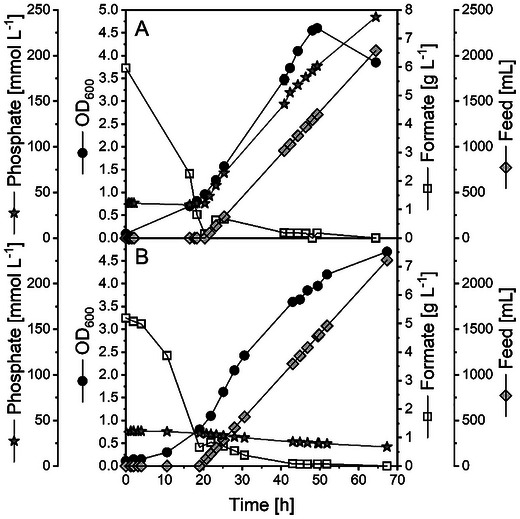
Formate fermentation process development with *M. extorquens* TK 0001. Upscaling of formate fermentation to a 10 L scale fed‐batch process in minimal media with (A) formate in 1 mol L^−1^ KPi buffer and (B) formate in water (phosphate‐depleted conditions). In both processes shown in A and B, the pH was titrated with H_2_SO_4_. Data acquired in individual experiments.

To study the effect of phosphate salts on growth in more detail in a controlled environment, a new set of fed‐batch experiments was carried out in a 10 L bioreactor. Two feeding solutions were compared to identify the influence of phosphate concentration on the process performance. The first feeding solution was composed of 1.0 mol L^−1^ KPi catholyte with 50 g L^−1^ of commercial formate in MO medium solution A (Figures 5A and S5C). Subsequently, fermentation was run with a feeding solution of 50 g L^−1^ of commercial formate in MO medium solution A (phosphate‐depleted conditions; see Table S2 for MO medium composition) (Figures 5B and S5 D). In both cases, sulfuric acid was used for pH titration instead of phosphoric acid.

Again, the cells grew from early on in the batch phases and consumed formate rapidly. The feeding rate was controlled to maintain a stable formate concentration in the fermentation media below 1 g L^−1^ throughout the whole process time. The determined Y_X/S_ in the batch phase was in a similar range compared to the 2 L scale fermentations (44–46 mg_CDW_ g_formate_^−1^). Again, an improved Y_X/S_ was observed in the feed phases of both experiments. However, a sudden drop in OD was found after about 50 h in the experiment with a feed of phosphate‐containing substrate solution (Figure 5A), consistent with the 2 L fermentation of eCO2R‐derived formate solution (Figure 4B). Consequently, biomass yield Y_X/S_ was improved to 107 mg_CDW_ g_formate_^−1^ when the phosphate‐depleted condition was used (Figure S5B). This means an improvement by about 5 % compared to the phosphate‐containing catholyte.

In addition, the process with phosphate‐depleted formate solutions showed a positive scale‐up behavior, with achieved biomass yields Y_X/S_ in the 10 L fermentation substantially enhanced by 35 % compared to the corresponding 2 L experiment, indicating good applicability for further scale‐up (Table 1).

**TABLE 1:** Comparison of performance indicators from fermentation experiments in 2 L and 10 L bioreactors.

	2 L scale		10 L scale	
	Commercial formate	eCO2R formate	Standard conditions	Phosphate‐depletion
**μ_batch_ [h^−1^]**	0.08	0.09	0.09	0.10
**Y_X/S, batch_ [mg_CDW_ g_formate_^−1^]**	54.00	52.00	44.00	46.00
**q_S, batch_ [g_formate_ g_CDW_^−1^ h^−1^]**	1.55	1.80	2.14	2.17
**μ_fed‐batch_ [h^−1^]**	0.03	0.03	0.04	0.04
**Y_X/S, fed‐batch_ [mg_CDW_ g_formate_^−1^]**	101.00	79.00	101.00	107.00
**q_S, fed‐batch_ [g_formate_ g_CDW_^−1^ h^−1^]**	0.33	0.37	0.34	0.36
**Final CDW concentration [g_CDW_ L^−1^]**	0.73	1.00	1.19	1.40

Legend: μ; specific growth rate, Y_X/S_; biomass‐substrate yield, q_S_; specific substrate uptake rate, CDW; cell dry weight.

Our results show clearly a negative effect of increasing phosphate concentrations on *M. extorquens* growth and Y_X/S_. This is reflected by the reduced OD_600_ after 30 h of fermentation and the drop in the optical density (from 4.6 to 3.9) at the end of the process in the fed‐batch fermentation using the phosphate‐containing catholyte (Figure 5A). In comparison, the phosphate‐depleted condition showed a more stable growth until the end of the process with a final optical density of 4.7 (Figure 5B). Finally, the process using phosphate‐depleted conditions can be maintained for a prolonged time indicating that even higher biomass concentrations are achievable when phosphate concentration is kept at low level (Figure 5B). This has to be balanced with a minimum phosphate concentration in the electrolyte to facilitate sufficient conductivity in the electrochemical CO_2_ reduction.

### 2.6 **Evaluation of Ene**rgy Efficiency

To comprehensively compare the effect of the KPi electrolyte solution during *M. extorquens* fermentation, we assessed the energetic efficiency for substrate conversion into biomass (E_aer_ / %), as per Equation 1 [36]. Energy efficiency was evaluated across both batch and fed‐batch phases under 2 and 10 L scale bioreactors. Required values of combustion energy were retrieved ref. [34], that is –20 kJ g_CDW_^−1^ for biomass combustion and –245 kJ mol^−1^ for formic acid (substrate) combustion. For more detailed information regarding the calculation (Equation 1) and underlying assumptions and parameters, the reader is referred to ref. [34].

(1) $$\;E_{\mathrm{aer}}=Y_{\mathrm{biomass}}\;x\;\Delta G_{\mathrm{comb}}^{\mathrm{biomass}}\Delta G_{\mathrm{comb}}^{\mathrm{biomass}}/\Delta G_{\mathrm{comb}}^{\mathrm{substrate}}\Delta G_{\mathrm{comb}}^{\mathrm{substrate}}$$

**FIGURE 6:**
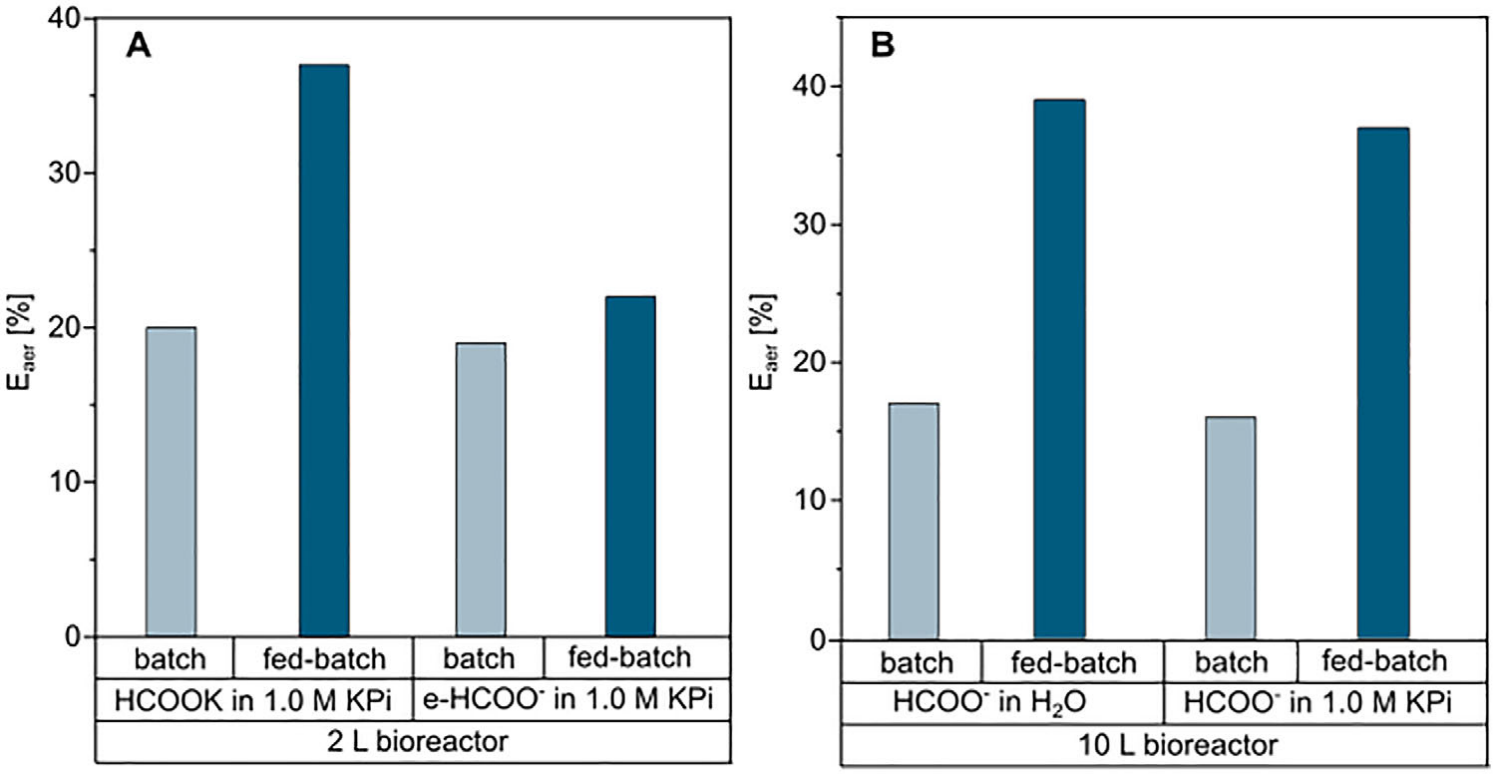
Estimated process energy efficiency of the developed bioprocess with *M. extorquens* TK 0001. Calculated energetic efficiency at (A) 2 L scale bioreactor and (B) 10 L scale bioreactor.

The bioprocess in the 2 L scale bioreactor revealed a negative impact of feeding electrochemically derived formate during the fed‐batch phase, with a 40 % decrease in efficiency compared to the process on commercial potassium formate (Figure 6A). Those results suggest an inhibitory component formed during electrolysis that impacts microbial performance [48].

The individual effect of phosphate‐containing electrolyte was further evaluated in a 10 L bioreactor (Figure 6B). Our results revealed that limiting phosphate concentration by, for example, pH adjustment with a phosphate‐free acid improves process efficiency. In the fed‐batch operation mode, E_aer_ was around 40 % for formate in water and in 1.0 M KPi. Furthermore, considering that organic acids are taken up by microbial cells in the protonated form, formate ions assimilation by the cells leads to proton removal from the medium, resulting in a pH increase (Figure 3A). Thus, the presence of 1.0 M KPi creates a buffer system controlling the pH and reducing external pH adjustments. These findings align with our group's recent work comparing *M. extorquens* growth on formate in phosphate buffer versus pure formic acid feedstocks [20]. Our previous studies demonstrated optimal growth when formic acid served as both carbon source and pH titration agent, achieving an E_aer_ of 30 % during fed‐batch operation [20]. Finally, the similar energy efficiencies calculated for both 2 and 10 L scales demonstrated the process robustness for scale‐up.

## 3 Conclusions

We successfully demonstrated the integration of electrochemical CO_2_ reduction to formate with microbial fermentation using *Methylorubrum extorquens* TK 0001. Our strategy was grounded in a rational design of the two‐step process cascade, with particular emphasis on selecting a buffer system that not only functions as an electrolyte during CO_2_ reduction but is also compatible with the fermentation process. In this setup, the electrolyte containing dissolved formate serves as the feed stream for fermentation. Through systematic screening and optimization, potassium phosphate was identified as the optimal buffer for *M. extorquens*, supporting both robust electrochemical CO_2_ reduction to formate and effective microbial growth. High formate concentrations were attainable either through extended electrolysis (48 h) or by employing electrode reactivation with electrolyte refreshment cycles lasting 2 to 4 h. Upon transferring the electrochemically generated formate to the bioreactor, meticulous control of phosphate concentration in the reactor medium proved critical; phosphate concentration had to be limited to a maximum of 0.1 M to achieve high biomass yields.

Remarkably, by precisely regulating the medium's phosphate concentration, we increased the biomass yield of *M. extorquens* on formate in fed‐batch fermentation by over 94 % compared to pure batch processes, reaching up to 107 mg_CDW_ g_formate_^−1^. This yield exceeds the values reported for engineered, state‐of‐the‐art synthetic formatotrophs [21, 22, 47]. Furthermore, the developed process was successfully scaled up from 2 L to a 10 L bioreactor using eCO2R‐derived formate, while maintaining comparable performance parameters.

To the best of our knowledge, this study constitutes the first rational development of a *combined* process involving (a) the electrosynthesis of formate from CO_2_ and (b) formatotrophic fermentation (here with *M. extorquens*) using solely electrochemically generated, formate‐rich electrolyte solutions. While previous studies have established the feasibility of using electrochemically derived formate as a substrate for formatotrophic fermentation, our work distinguishes itself through the targeted optimization of the integrated process cascade, particularly at the interface between the two steps. In this context, the selection of the electrolyte for efficient electrochemical formate production substantially influences downstream fermentation performance. Fine‐tuning the electro‐biochemical process interface is essential for establishing a high‐performing, integrated production system and is demonstrably more effective than the independent optimization of each step, as is commonly practiced in research. Thus, our findings provide a practical framework for the design of integrated electro‐biochemical cascade processes aimed at sustainable CO_2_ utilization. Looking ahead, the advancement of compatible and robust electrochemical and fermentation processes will unlock versatile opportunities for producing value‐added chemicals using CO_2_ as a feedstock.

## 4 Experimental Section

### 4.1 Materials and Solutions Preparation

Dipotassium phosphate (K_2_HPO_4_, 99 %, Th. Geyer), monopotassium phosphate (KH_2_PO_4_, ≥99 %, Roth), tris‐(hydroxymethyl)‐amino methane (TRIS, C_4_H_11_NO_3_, ≥99.3 %, Roth), sodium bicarbonate (NaHCO_3_, ≥99 %, Roth), sodium carbonate (Na_2_CO_3_, ≥99.5 %, Roth), citric acid (C_6_H_8_O_7_, ≥99.5 %, Roth), potassium bicarbonate (KHCO_3_, 99.7 %, Sigma–Aldrich), hydrochloric acid (HCl, 37 %, Sigma–Aldrich), and 5 % Nafion (Sigma–Aldrich) were used as received. CO_2_ gas (99.995 %) was supplied by Linde (Germany). The composition of the biological buffers used throughout this work is detailed in Table 2.

**TABLE 2:** List of biological buffers used in this work.

Buffers	Abbreviation	Composition	Conductivity [mS cm^−1^]	pH (initial) [‐]
Potassium hydrogen carbonate	KHCO_3_	—	72.6	8.06
Carbonate buffer	NaCBi	Na_2_CO_3_/NaHCO_3_	52.9	8.54
Phosphate Buffer	KPi	K_2_HPO_4_/KH_2_PO_4_	99.7	7.56
Citrate buffer	CPi	C_6_H_8_O_7_	40.4	3.32
Trisaminomethane	TRIS	C_2_H_11_NO_3_	35.7	7.43

### 4.2 Electrode Preparation

A suspension of Sn nanoparticles (<150 nm, Sigma–Aldrich) and activated carbon prepared in a mixture of distilled water, isopropanol, and 5 % Nafion was sonicated for 30 min. This catalyst ink was drop‐coated onto a gas diffusion layer (GDL, 10 cm^2^, Freudenberg H23C6) at 80 °C and stored overnight in air. The final catalyst load was 10 mg cm^−2^.

### 4.3 Electrochemical Measurements

Electrochemical experiments were conducted in a custom‐designed 2‐compartment flow cell (Electrocell, Denmark) using Sn gas diffusion electrode (GDE) as the working electrode, Ir‐MMO (Iridium Mixed Metal Oxide) as the counter electrode, and Ag/AgCl (eDAQ, Denmark) as the reference at room temperature. A cation exchange membrane (CEM, Fumasep, Fumatech, Germany) separated the anodic and cathodic cell compartments. Electrolyte flow was maintained at 100 mL min^−1^ (Watson–Marlow 323 pump) with CO_2_ gas supplied at 50 mL min^−1^ (Brooks Instrument mass flow controller). The potentiostat (PGSTAT204, Metrohm, Switzerland) was controlled by NOVA 2.1 software (Metrohm, Switzerland). pH and conductivity were monitored using VWR pH 3210 and pHenomenal CO 3100H meters.

### 4.4 Bacterial Strains and Cultivation Routines

*Methylorubrum extorquens* TK 0001 DSM 1337 (DSMZ, www.dsmz.de) was grown in MO medium prepared from sterile stock solutions. For strain maintenance, exponentially growing cells from liquid cultures were harvested, mixed with sterile glycerol (final glycerol concentration of 40 % (v/v), frozen, and stored at −80 °C (Revco CxF ‐86C Chest Freezers, Thermo Fisher Scientific, United States). MO medium recipe and preparation was conducted as described previously [49]. Strain propagation was carried out in MO medium mixed with 18 g L^−1^ Agar–Agar Kobe I.

To obtain viable cells for cultivation in shake flasks (e.g. to determine buffer effects or phosphate toxicity on growth) or parallelized microbioreactors, a first preculture of *M. extorquens* was inoculated in 50 mL MO medium containing 0.5 % methanol (v/v) in 250 mL baffled shake flasks using a single colony from a MO agar plate before incubation at 30 °C, 150 rpm for 72 h (New Brunswick Innova 44, Eppendorf SE, Germany). Subsequently, a second preculture of 50 mL MO medium containing 0.5 % methanol (v/v) in 250 mL baffled shake flasks was inoculated to an initial OD_600_ of about 0.05 (Ultrospec 10, Biochrom Ltd, United Kingdom) using viable cells from the first preculture before incubation at 30 °C, 150 rpm for 24 h. For fermentation in bioreactors, precultures were prepared as described, using MO medium with 1 g L^−1^ of potassium formate and 1 % (v/v) methanol.

Main cultures, either for shake flask, parallelized microbioreactor or lab‐scale bioreactor experiments, were inoculated and handled as described in the following sections. In the case of buffer effect and phosphate tolerance screening cultivations in shake flasks, the pH was titrated at 24 h to pH ∼7 using 1 M HCl.

To examine the influence of the electrochemical buffers on growth of *M. extorquens*, the minimal medium was supplied with 100 mM of either KHCO_3_, NaCBi, KPi, TRIS, or CPi buffers (pH 6.8). The same was done when the KPi buffer or formate concentration effect on growth of *M. extorquens* was determined: Increasing concentrations of up to 1 M of KPi (pH 6.8) were used.

All cultivations (shake flasks or lab‐scale bioreactors) were handled using the same procedure: On a regular basis, cultivation broth samples were withdrawn from the cultivation vessels and the OD_600_ was measured. Next, the samples were centrifuged (Multifuge X3R, Thermo Fisher Scientific, 4200 x g, 5 min, 20 °C) and the supernatants were separated and stored for following analytical procedures.

### 4.5 Batch Cultivation in Parallelized Microbioreactors

Substrate tolerance of *Methylorubrum extorquens* TK0001 was investigated by cultivating the strain with increasing formate concentrations in a high‐throughput experiment using a parallelized and miniaturized microbioreactor system (1‐2 mL scale) (BioLector XT, Beckman Coulter GmbH, Germany).

The main cultures were inoculated in 800 µL MO media to an initial OD_600_ of 0.05 using washed exponentially growing cells from the second preculture. To remove methanol residues, the second preculture was treated as follows. First, centrifugation was performed at 4200 g for 5 min at 20 °C (High speed centrifuge Avanti J‐E Series, Beckman Coulter GmbH, Germany). The supernatant was discarded, and the pellet was resuspended in 50 mL sterile MilliQ water. The washing step was carried out twice.

To determine the tolerance limit, the media contained varying formate concentrations (g L^−1^). Potassium formate was added from a pH neutralized stock solution of 336.48 g L^−1^. Standard conditions without formate were applied as the negative control.

Cultivation was conducted at 1200 rpm and 30 °C under atmospheric air composition (20.95 % O_2_) and humidity set to 85 %. Cell density was recorded by acquisition of the backscatter signal at 620 nm. The pH was adjusted to 6.8 using microfluidics with 2 mol L^−1^ H_2_SO_4_ and 3 mol L^−1^ NaOH. Following the end of fermentation, the offline OD_600_ of broths representing the respective individual process conditions was measured for correlation with backscatter measurements. All main cultivations were performed in biological duplicates.

### 4.6 Fed‐Batch Cultivation in Stirred Tank Bioreactors

Formate fed‐batch cultures were cultivated in 2 and 10 L stirred tank bioreactors (BIOSTAT B plus 2 L and BIOSTAT C plus 10 L, Sartorius AG, Germany) with minimal medium. Exponentially growing precultures were used for inoculation at OD_600_ = 0.1. A linear formate feeding was initiated when formate depletion approached, with pH (pH, EasyFerm Bio HB K8 120, Hamilton, Swiss) kept at 6.8 ± 0.1 by automatic phosphoric acid or sulfuric acid titration. Dissolved oxygen (pO_2_, OxyFerm FDA 120, Hamilton, Swiss) was controlled by stirrer speed and aeration rate adjustments.

To start the fermentation process, a calculated volume of exponentially growing preculture broth was directly taken to inoculate the fermentation to an initial OD_600_ of 0.1. The initial volume of the process was 700 mL (2 L fermentation) or 6 L (10 L fermentation). The standard minimal medium was used as the initial batch medium, supplemented with 5 g L^−1^ potassium formate or 5 g L^−1^ electrochemically derived formate in 1 mol L^−1^ potassium phosphate buffer. During fermentation, samples were collected regularly for measuring OD_600_, pH, and substrate quantification. When depletion of initially supplied formate was approaching, the feed phase in 2 L scale was initiated by feeding a solution of 65 g L^−1^ potassium formate (commercial or electrochemically derived) in 1 mol L^−1^ potassium phosphate buffer (pH 6.8) (i.e., the electrolyte in case of electrochemically produced formate). To avoid carbon depletion during the feed phase and maintain carbon limitation, a linear feed was set, calculated to satisfy cellular substrate demand, and keep formate concentration well below 1 g L^−1^. In 10 L scale the influence of phosphate in the feed solution on growth of *M. extorquens* was examined by using 50 g L^−1^ commercial potassium formate in 1 M phosphate buffer (pH 6.8) or 50 g L^−1^ potassium formate. During the process, the temperature was kept at 30 °C ± 0.2 °C, and a pO_2_ of 20 % was maintained by applying a cascaded control at first increased stirrer speed followed by an increase of aeration rate. In detail, the initial stirrer speed was set to 250 rpm (maximum 1000 rpm) with an initial aeration rate of 0.06 sL h^−1^ (maximum 3 sL h^−1^). Data acquisition and process control were carried out by BIOSTAT software.

### 4.7 Analytical Methods

Formate and phosphate concentrations in catholyte and fermentation broths were analyzed by HPLC with a UV‐Vis detector (λ = 210 nm) and RI detector (Shimadzu, Japan), equipped with a Rezex ROA‐Organic Acid H+ (8 %) column (Phenomenex, Germany). The isocratic method was performed with 0.005 N H_2_SO_4_ as eluent at 65 °C and 0.5 mL min^−1^ flow rate. Data were used to calculate Faradaic efficiency (FE), formation rate, and microbial substrate uptake rates. A summary of the calculations is available as Supporting Information.

### 4.8 Characterization Techniques

Surface morphology of the working electrode before and after electrolysis was analyzed with scanning electron microscopy (SEM) coupled with an energy‐dispersive X‐ray using a Zeiss DSM 940 A microscope.

## Conflicts of Interest

The authors declare no conflicts of interest.

## Supporting information




**Supporting File 1**: The Supporting Information includes additional data and more detailed information on electrochemical CO_2_ reduction, electrode characterization, fermentation, experimental procedures, and calculations, as well as a tabular comparison of our study to previously published studies with respect to various performance indicators.
**Supporting File**: chem70581‐sup‐0001‐SuppMat.docx.

## Data Availability

The data that support the findings of this study are available from the corresponding author upon reasonable request.
