# Supplementary material for: Electrocatalytic CO2 Reduction Coupled to Formate Fermentation: An Electro‐Bio Cascade Approach in Biocompatible Electrolytes
Source: Chemistry. 2025 Dec 24;32(7):e02658. doi: 10.1002/chem.202502658 (PMC12910421; doi:10.1002/chem.202502658)
Supplement: Supplementary file 1 — Supporting File 1: The Supporting Information includes additional data and more detailed information on electrochemical CO2 reduction, electrode characterization, fermentation, experimental procedures, and calculations, as well as a tabular comparison of our study to previously published studies with respect to various performance indicators. Supporting File: chem70581‐sup‐0001‐SuppMat.docx. [file CHEM-32-e02658-s001.docx]

**Electrocatalytic CO_2_ reduction coupled to formate fermentation: an electro-bio-cascade approach in biocompatible electrolytes**

Luciana Vieira^a,ǂ^, Jonathan Thomas Fabarius^a,ǂ^, Gabriela Piton ^a,b,ǂ^, Barbara Bohlen^a,b,^, Dhananjai Pangotra^a^, Melanie Speck^a^, Carina Sagstetter^a^, Volker Sieber^a,b^, Arne Roth^a,^ *

^ǂ^*Equal contribution*

**Email:* [*arne.roth@igb.fraunhofer.de*](mailto:arne.roth@igb.fraunhofer.de)

*^a^Fraunhofer Institute for Interfacial Engineering and Biotechnology (IGB), BioCat Branch for Bio, Electro and Chemocatalysis, Schulgasse 11a, 94315 Straubing, Germany*

*^b^TUM Campus Straubing for Biotechnology and Sustainability, Technical University of Munich, Schulgasse 16, 94315 Straubing, Germany*

Table of contents

[Calculations 2](#_Toc214991692)

[Screening of electrolyte solutions 4](#_Toc214991693)

[Optimization of current density 5](#_Toc214991694)

[Continuous electrosynthesis of formic acid 6](#_Toc214991695)

[Biomass yield in microbial formate fermentation 8](#_Toc214991696)

[Minimal medium composition 9](#_Toc214991697)

[Literature review on electrochemical CO2 conversion to formate 11](#_Toc214991698)

[References 12](#_Toc214991699)

## Calculations

The **electrochemical formation rate of formate (𝜉)** was determined according to Equation 1, where the number of moles of formic acid (n) measured experimentally was divided by the electrode's geometric area (A) in cm², and the corresponding measurement time (t) in h:

| $\xi=\frac{n}{A \times dt}$ |  | (1) |
| --- | --- | --- |

To determine the **Faradaic efficiency (FE)**, the theoretical formic acid production for the total applied charge (Q_total_) was divided by the actual charge required for producing the detected formic acid concentration (Q_formate_). The charge of the formic acid production was computed by multiplying the number of moles of formic acid (*n*) detected in the electrolyte with the number of electrons required for the CO_2_R to formic acid (*z*) and the Faraday constant (*F*) as given in Equation 2:

| $FE= \frac{Q_{formate}}{Q_{total}}\times100\%= \frac{n\times z\times F}{Q_{total}}\times100\%$ |  | (2) |
| --- | --- | --- |

The **energy consumption (EC)** for producing formic acid was determined based on the number of moles of formic acid (*n*) generated:

| $EC= \frac{Q_{total}\times E_{cell}}{n}\times2.28 ․ {10}^{-4}$ |  | (3) |
| --- | --- | --- |

In Equation 3, Q_total_ represents the total charge that has passed through the electrochemical cell, E_cell_ denotes the cell potential in volts, and 2.28 x 10^-4^ is the conversion factor for units from Ws·mol^-1^ to kWh·kmol^-1^.

The electrochemical process Energy Efficiency (EE) was calculated using Equation 4 and indicates how efficiently the total energy supplied was used for the production of formic acid. E0 is the standard reduction potential for the reduction reaction of CO2 to formic acid (-0.2 V vs. RHE) and E is the potential of the cathode.

$EE= \frac{E^{0}}{E} \times FE$ (4)

To quantify **microbial growth rate** the time-dependent change of optical density was used to infer the growth rate µ (1 h^‑1^). Equation 5 shows the used calculation.

$\mu=\frac{ln(\frac{{OD}_{600,t1}}{{OD}_{600, t0}})}{t1-t0}$ (5)

In order to quantify **biomass yield (Y_x/s_)** (i.e. amount of biomass formed per mass of substrate), the change in CDW concentration was plotted against change in substrate concentration. The obtained slope of the correlation is defined as the biomass yield (Y_X/S_, [g_CDW_ g_Substrate_]) (Equation 6).

$Y_{X/S}=\frac{\Delta CDW}{\Delta\left[ c_{Substrate} \right]}$ (6)

The optical density (OD600) at 600 nm (Ultrospec® 10 (RS232), Biochrom Ltd., Cambridge, United Kingdom) was utilized as a measure for cell concentration. The cell dry weight (CDW) was inferred from the optical density using Equation 7. The correlation factor used was determined by weighing dried biomass samples obtained from exponentially growing cells.

0.305 g_CDW_ L^-1^ OD_600_^-1^  (7)

## Screening of electrolyte solutions

**Figure S1.** Screening of electrolyte solutions. A) Energy consumption in kWh kmol^-1^; B) Energy efficiency in %. Experimental conditions: Sn-NPs catalyst (10 mg cm^-2^); Current density: - 100 mAcm^-2^; Electrolyte: 1.0 mol L^-1^ KPi pH 7.5; 2 hours electrolysis. Electrochemical experiments were conducted in two independent replicates.

## Optimization of current density

To examine the influence of current density on formic acid production, a galvanostatic screening was conducted across a current density range of -50 to -400 mA cm^-2^ . Formic acid concentration was analyzed after 30 minutes of electrolysis at constant current, and the data was used to derive FE, formic acid formation rate, energy consumption, and energy efficiency.

Formic acid concentration increased with current density up to 64 mmol L^-1^ at -150 mA cm^-2^; at higher current densities, formic acid concentration decreased consistently with less than 4.0 mmol L^-1^ produced at -400 mA cm^-2^. A similar trend was observed with FE, except that the highest FE was calculated at -100 mA cm^-2^ (76 %). Another relevant factor is the energy efficiency of formic acid production. Since EE has a direct relationship with FE, they follow the same trend. Therefore, formic acid production EE reached a maximum value of 15.4 % at -100 mA cm^-2^, and it constantly decreased at higher current densities.

**Figure S2.** Optimization of current density. A) Faradaic efficiency; B) Formic acid concentration; C) Formic acid formation rate; D) Energy consumption; E) Energy efficiency. Experimental conditions: Sn-NPs catalyst (10 mg cm^-2^); Electrolyte: 1,0 mol L^-1^ KPi pH 7.5; 30 minutes electrolysis.

## Continuous electrosynthesis of formic acid


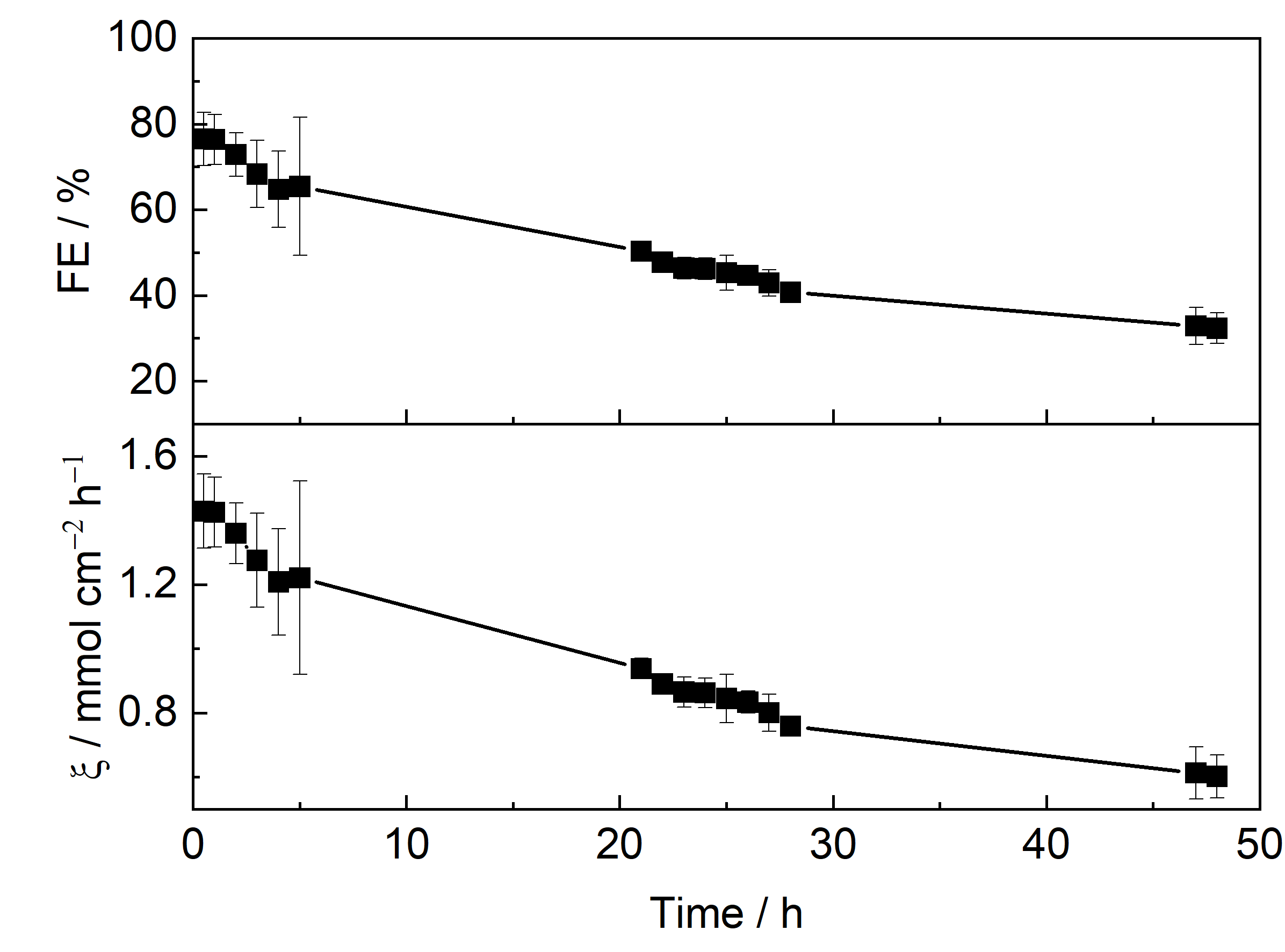


**Figure S3.** Calculated Faradaic efficiency and formic acid formation rate over a 48h electrolysis at -100 mA cm^-2^ in 1.0 mol L^-1^ KPi buffer pH 7.5. Electrochemical experiments were conducted in four independent replicates.


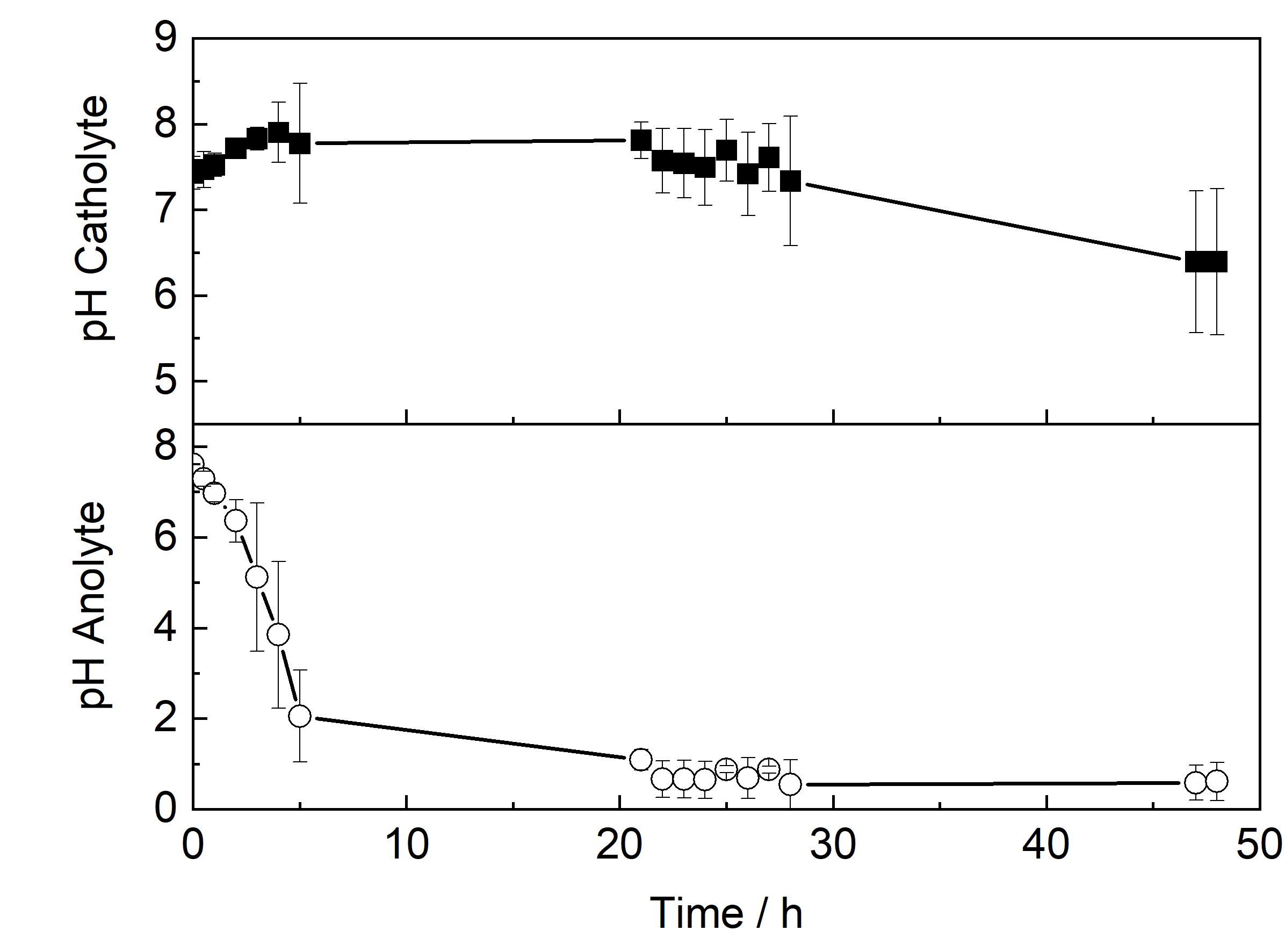


**Figure S4.** pH variations of catholyte and anolyte solutions over a 48h electrolysis at – 100 mA cm^-2^ in 1.0 mol L^-1^ KPi buffer pH 7.5. Electrochemical experiments were conducted in four independent replicates.


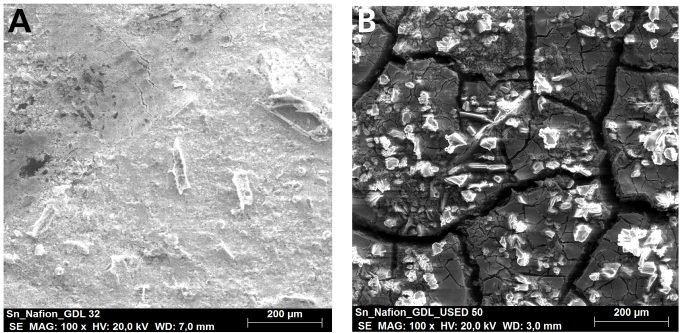


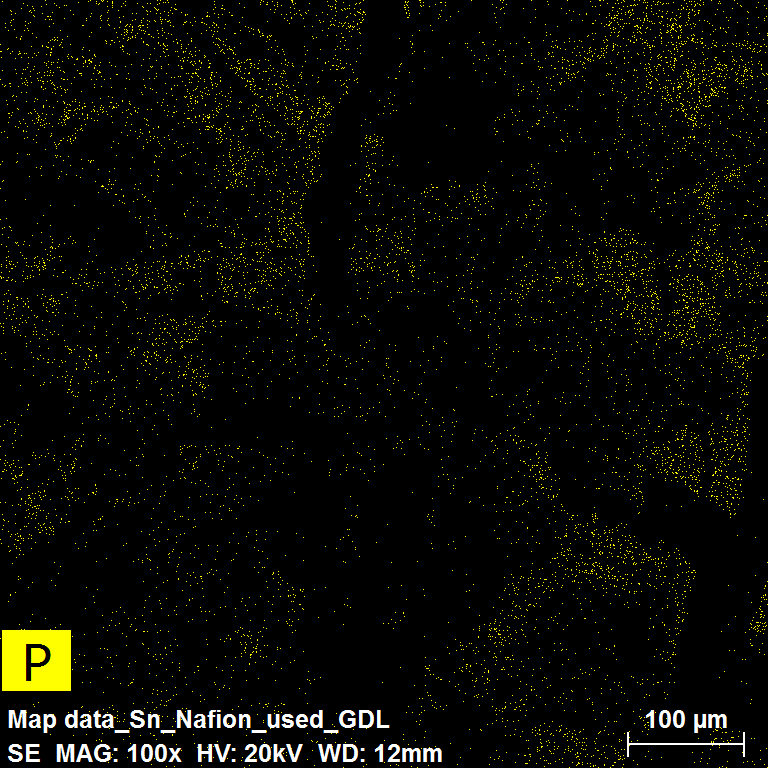
**Figure S5.** Surface morphology characterization by scanning electron microscopy of working electrode (A) before and (B) after 24 hours electrolysis.

B

A


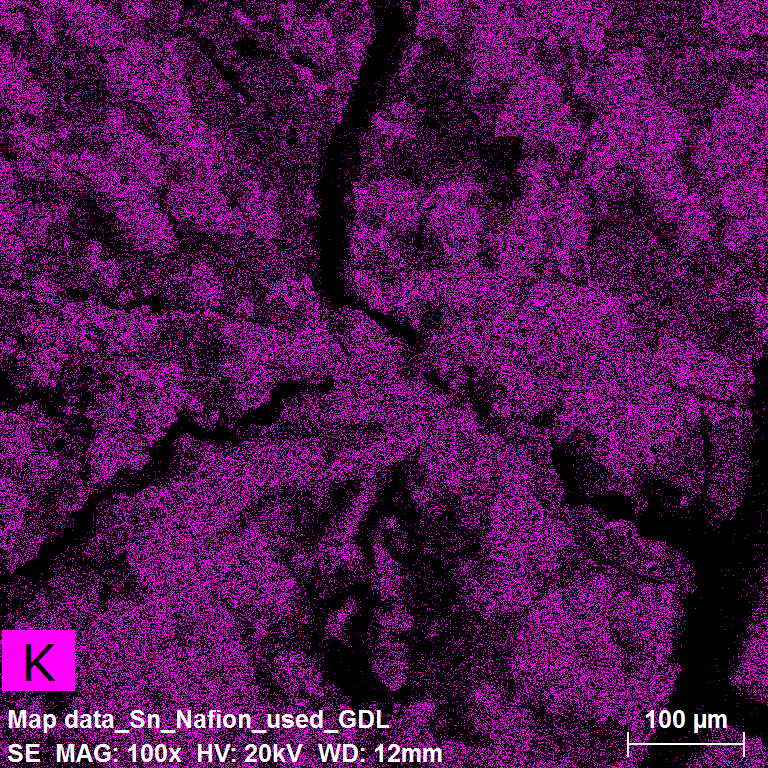


**Figure S6.** Energy-dispersive X-ray (EDX) characterization of working electrode (cathode) after 24 hours of electrolysis. (A) potassium mapping, (B) phosphorus mapping.

## Biomass yield in microbial formate fermentation


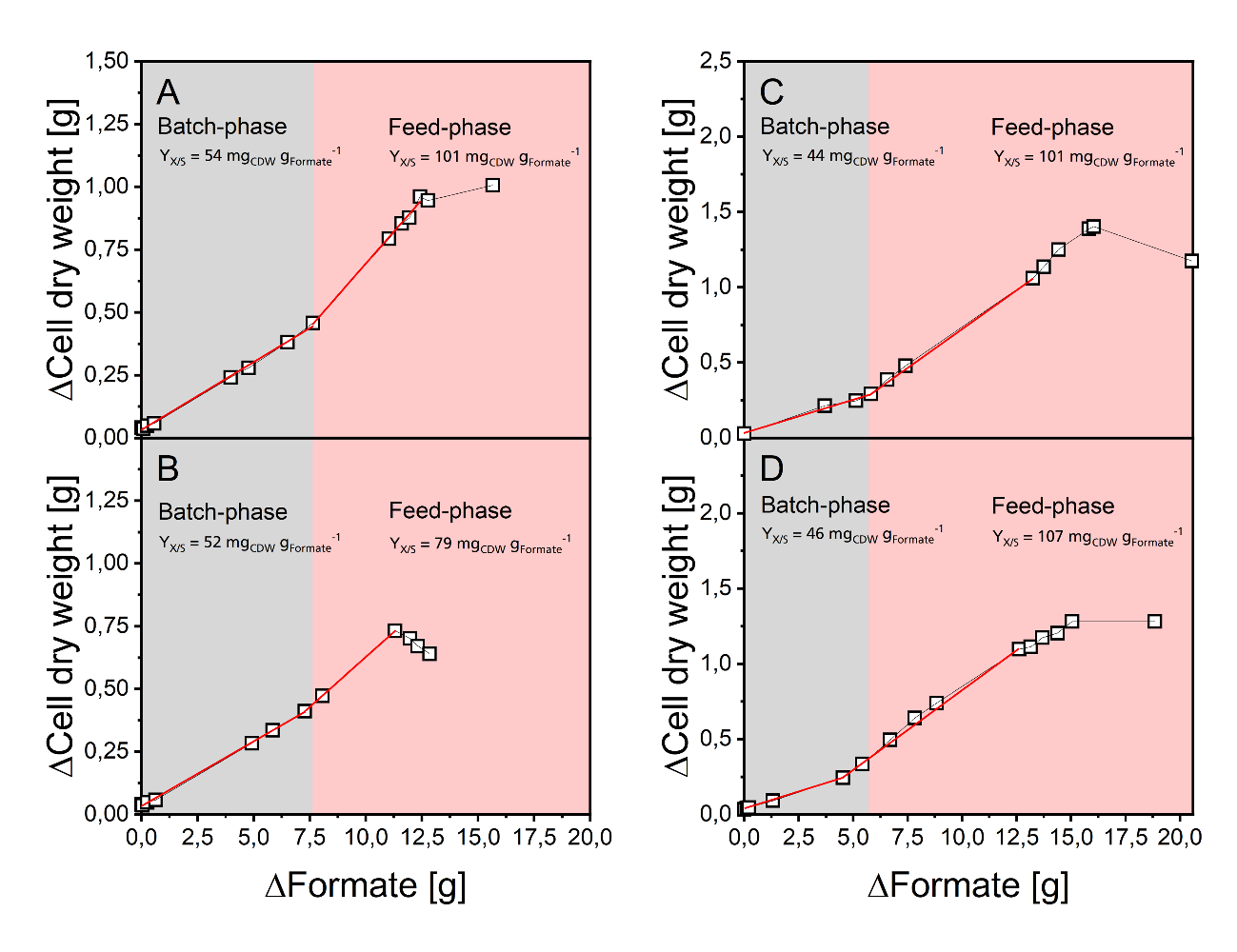


**Figure S7**. Biomass-substrate yield (Y_X/S_) determination of formate fermentation. Shown is the plot correlating cell dry weight to consumed formate in 2 L scale fermentation using (A) commercial formate and (B) eCO2R formate or 10 L scale fermentation using (C) eCO2R formate feeding solution and (D) phosphate-depleted commercial formate solution.

## Minimal medium composition

**Table S1.** List of chemicals used throughout the study and the suppliers

| **Chemical** | **Supplier** |
| --- | --- |
| Agar agar Kobe I, powder for microbiology | Carl Roth GmbH + Co KG, Germany |
| Ammonium molybdate, 99.98% | Sigma-Aldrich Chemie GmbH, Germany |
| Ammonium sulfate, ≥99.5% | AppliChem GmbH, Germany |
| Boric acid, ≥99.5% | Sigma-Aldrich Chemie GmbH, Germany |
| Calcium chloride dihydrate, 99% | Merck KGaA, Germany |
| Cobalt-(II)-chloride hexahydrate, ≥99% | Carl Roth GmbH + Co KG, Germany |
| Copper-(II)-sulfate, ≥99% | AppliChem GmbH, Germany |
| Di-sodium hydrogen phosphate dihydrate, ≥99% | AppliChem GmbH, Germany |
| Glycerol Electran®, ≥98% for molecular biology | VWR International GmbH, Germany |
| Hydrochloric acid fuming 37%, AnalaR NORMAPUR® ACS | VWR International GmbH, Germany |
| Iron-(II)-sulfate heptahydrate, ≥96% | VWR International GmbH, Germany |
| Magnesium sulfate heptahydrate, 99.5% | AppliChem GmbH, Germany |
| Manganese-(II)-chloride tetrahydrate, for analysis, EMSURE® ACS | Merck KGaA, Germany |
| Methanol ROTISOLV®, ≥99% | Carl Roth GmbH + Co KG, Germany |
| Potassium dihydrogen phosphate ≥99.5% AnalaR | VWR International GmbH, Germany |
| Potassium formate, 99% | Sigma-Aldrich Chemie GmbH, Germany |
| Sodium citrate tribasic dihydrate, ≥99% | Sigma-Aldrich Chemie GmbH, Germany |
| Sodium hydroxide, ≥98% | Carl Roth GmbH + Co KG, Germany |
| Sodium tungstate dihydrate, ≥99% | Sigma-Aldrich Chemie GmbH, Germany |
| Sulfuric acid, ROTIPURAN®, 98 % | Carl Roth GmbH + Co KG, Germany |
| Zinc sulfate heptahydrate, 98 % | Thermo Fisher Scientific, United States |

**Table S2.** MO media recipe adapted from L.-Y. Cui et al., 2016^[1]^

|  | **Composition** | **Molecular weight**  **[g mol^-1^ ]** | **End concentration  MO minimal medium** | | **Note** |
| --- | --- | --- | --- | --- | --- |
| **Solution A** | (NH_4_)_2_SO_4_ | 132.14 | 7.57 mM | Mixed, steam sterilization,121 °C, 20 min | |
|  | MgSO_4_ x 7 H_2_O | 246.47 | 1.82 mM |  |  |
|  | CaCl_2_ x 2 H_2_O | 147.02 | 0.022 mM |  |  |
| **Solution B** | Na_3_C_6_H_3_O_7_ x 2 H_2_O | 294.10 | 25.08 µM | Mixed, pH adjusted to 1.0 with 37 % (v/v) HCl, sterilization by filtration, 0.2 µm PES filter | |
|  | ZnSO_4_ x 7 H_2_O | 287.54 | 0.66 µM |  |  |
|  | MnCl_2_ x 4 H_2_O | 197.90 | 0.55 µM |  |  |
|  | FeSO_4_ x 7 H_2_O | 278.01 | 9.90 µM |  |  |
|  | (NH_4_)_6_Mo_7_O_24_ x 4 H_2_O | 1235.86 | 1.10 µM |  |  |
|  | CuSO_4_ x 5 H_2_O | 249.68 | 0.55 µM |  |  |
|  | CoCl_2_ x 6 H_2_O | 237.93 | 1.10 µM |  |  |
|  | Na_2_WO_4_ x 2 H_2_O | 329.85 | 1.18 µM |  |  |
| **Solution C** | H_3_BO_3_ | 61.83 | 0.485 µM | Sterilization by filtration, 0.2 µm PES filter | |
| **Solution D** | KH_2_PO_4_ | 136.09 | 14.87 mM | Mixed, steam sterilization,121 °C, 20 min | |
|  | Na_2_HPO_4_ x 2 H_2_O | 177.99 | 23.25 mM |  |  |
| **Solution E** | CH_3_OH | 32.04 | varies | Sterilization by filtration, 0.2 µm PTFE filter | |
| **Solution F** | CHKO_2_ | 84.12 | varies | pH adjusted to 7.0 with 10 mol L^-1^ KOH, steam sterilization,121 °C, 20 min | |

## Literature review on electrochemical CO2 conversion to formate

**Table S3.** Comparison of electrochemical CO2 conversion to formate processes conducted in biologically compatible media and the respective biocatalyst for biochemical use of formate

| **Electrocatalysis** | | | | | **Biocatalysis** | | **Ref.** |
| --- | --- | --- | --- | --- | --- | --- | --- |
| **Catalyst** | **Electrolyte** | **[HCOO-] [mol L^-1^]** | **Current density [mA cm^-2^]** | **FE [%]** | **Biocatalyst** | **Main fermentation product and max concentration** |  |
| **Sn** | **0.5 M KPi** | **2.0** | **100** | **> 80** | ***M. extorquens* TK0001** | **Biomass / 0.73 g_CDW_ L^-1^** | **This work** |
| Sn | 0.2 M NaH2PO4/K2HPO4 | 0.441 | 150 | 76.6 | *C. necator* WT (resting cells) | PHB / n.a. | ^[2]^ |
| Sn | Phosphate-enhanced MR medium (30 g/L KH2PO4) (without TMS)^a^ | 0.333 | 120 | 66 | *C. Necator* | PHB / n.a. | ^[3]^ |
| In | 30 mM carbonate buffer | 0.0061 | - | 12.9 | *M. extorquens* AM-1 | 7µM mesaconate + 10µM 2S-methylsuccinate | ^[4]^ |
| In-NPs | Modified K2HPO4/ K2SO4 buffer^b^ | 0.01^c^ / 0.0201^d^ / 0.0168^e^ | 10 | 86 | *C. necator* H16 and *M. extorquens* AM-1 | PHB / *M. extorquens*: no PHB; *C. necator*: 13,0 mg/L (only when electricity was applied) | ^[5]^ |

^a^: TMS – trace metal solution; ^b^: 40 mM K2HPO4 + 100 mM K2SO4 + 3% (v/v) of medium C (cultures of *C. necator*) or M (cultures of *M. extorquens*), TMS and nitrogen-limited conditions, ^c^: 2 hours – media without bacteria, d: 2 hours in *C. necator* media, ^e^: 2 hours in M. extorquens media

## References

[1] L.-Y. Cui, W.-F. Liang, W.-L. Zhu, M.-Y. Sun, C. Zhang, X.-H. Xing, *Biochemical Engineering Journal* **2017**, *119*, 67-73.

[2] I. Dinges, I. Depentori, L. Gans, D. Holtmann, S. R. Waldvogel, M. Stöckl, *ChemSusChem* **2024**, *17*, e202301721.

[3] J. Lim, S. Y. Choi, J. W. Lee, S. Y. Lee, H. Lee, *Proceedings of the National Academy of Sciences* **2023**, *120*, e2221438120.

[4] R. Hegner, K. Neubert, C. Kroner, D. Holtmann, F. Harnisch, *ChemSusChem* **2020**, *13*, 5295-5300.

[5] I. S. Al Rowaihi, A. Paillier, S. Rasul, R. Karan, S. W. Grötzinger, K. Takanabe, J. Eppinger, *PLOS ONE* **2018**, *13*, e0196079.
